# The effect of landfill leachate treatment on ecotoxicological properties of *Folsomia candida*, with a focus on soil contamination risks

**DOI:** 10.1038/s41598-025-07799-5

**Published:** 2025-07-02

**Authors:** Dorota Szydełko, Aleksandra Wdowczyk, Iwona Gruss

**Affiliations:** 1 Department of Plant Protection, Wrocław University of Environmental and Life Sciences, pl. Grunwaldzki 24a, Wrocław, 50–363 Poland; 2 Department of Environmental Protection and Development, Wrocław University of Environmental and Life Sciences, pl. Grunwaldzki 24, Wrocław, 50–363 Poland

**Keywords:** Environmental monitoring, Pollution remediation

## Abstract

Landfilling is a common method for managing solid waste, but it generates leachate, a highly contaminated liquid that can threaten groundwater, surface water, and soil health. This study examines how raw and treated landfill leachate affects the survival and reproduction of *Folsomia candida*, a soil-dwelling Collembola species. Leachate treatment was conducted using Vegetation-activated sludge process with different fill materials (zeolite, biochar) and plant variations. The study also explored the relationship between leachate toxicity and its chemical properties to determine key contaminants influencing *F. candida*. The results showed that both raw and treated leachate reduced Collembola reproduction, with most treatments exhibiting toxicity levels similar to untreated leachate. The number of juveniles decreased by 49.8% with the biochar–plant system and by 64.9% with biochar. The number of adults decreased by 24.4% from day 7 to day 42. Redundancy analysis (RDA) revealed that iron (Fe) was strongly associated with increased mortality, while total dissolved solids (TDS) and organic nitrogen (ON) were positively linked to juvenile abundance. Although the treatment systems removed some contaminants, they were not fully effective, leaving residual pollutants that could pose environmental risks. These findings highlight the need for improved treatment methods to minimize leachate toxicity and protect soil ecosystems.

**Supplementary Information:**

The online version contains supplementary material available at 10.1038/s41598-025-07799-5.

## Introduction

Landfilling is one of the most widely used methods for managing solid waste^1^. However, this approach generates a highly polluted liquid known as leachate, formed through various physical, chemical, and biological processes within the landfill^2^including biodegradation of organic fraction, infiltration of precipitation and compaction of waste^3^. A particular threat is the migration of leachate, both from uncontrolled and organized landfills^4^. Leakage of leachate can cause contamination of groundwater, surface water, soils and natural ecosystems, especially when leachate is released in an uncontrolled manner^5,6^. Uncontrolled leakage of leachate may occur due to poor management, failure of leachate collection and treatment systems, or natural disasters^7^.

The composition of leachate depends on multiple factors, including the type of waste, moisture content, stage of decomposition, landfill design, geographical conditions, and water infiltration^8,9^. Common components of leachate include dissolved organic matter, inorganic nutrients, of ammonium nitrogen, sulphates, chlorides, suspended solids, dissolved organic matter, heavy metals, and synthetic organic chemicals^10^.

These pollutants present significant environmental and health challenges, as leachate toxicity is difficult to predict^11^. These substances pose threats to ecosystems, aquatic life, and food chains, with potential human health consequences including cancer, acute toxicity, and genetic damage^12,13^. Soil, as the foundational layer beneath landfills, is particularly vulnerable to leachate contamination. Without adequate sealing measures, such as geomembranes, leachate infiltration can severely degrade soil quality and alter its hydraulic properties^14^. This problem is particularly significant in developing countries, where most landfills do not have sealing measures, leachate collection, and treatment systems^15^. However, in most cases, landfills use barriers or barrier systems such as liners, covers and vertical walls to contain contaminants, preventing uncontrolled release of seepage into the environment and groundwater^16^. The studies conducted so far, covering landfills without leachate collection and treatment systems, have shown the influence of leachate on changes in the physicochemical and biological processes of the soil^17,18^. Moreover, the chemical interactions of leachate with soil can shift pH levels, increasing the solubility of harmful substances and posing risks to groundwater^13^.

Heavy metals from leachate, due to binding with soil particles, sediments or other matrices, may gradually accumulate over time, which is influenced by the physicochemical parameters of the soil^19,20^.

In recent years, many studies have been conducted on soil contamination mainly around open dumpsites/unlined municipal waste landfills. For example, Wang et al. (2020) found moderate to high risk of trace element contamination in soils around municipal waste landfills in Tibet, China. The highest Hg concentration of 0.015 mg/kg was found in soils surrounding landfills, which was 6 times higher than the background value^21^. Studies conducted in areas surrounding open dumpsites in Morocco showed that the average concentrations of Zn, Cd, Fe, Cu, Ni, Pb, and Cr were well above their geochemical background, and the soils near the dumpsite had moderate to high levels of contamination, mainly due to high Cd and Pb contents^22^. Many studies have highlighted that soil contamination is a significant problem, especially for people living near municipal waste dumps. The threat is not only from contact with contaminated soil, inhalation of particles, but also from eating contaminated food crops^19,23^. Given the risks of leachate infiltration, soil health assessment has become a crucial aspect of environmental management.

Another important issue is the discharge of already treated leachate into water or soil, which requires a comprehensive assessment of their properties. Considering that physicochemical analyses may not detect some pollutants present in leachate, it was proposed to supplement them with a toxicological assessment using biological tests with model organisms^12^.

Collembola, soil-dwelling arthropods, are valuable bioindicators of soil quality due to their sensitivity to contamination. They are effective indicators in environments affected by hydrocarbons and landfill restoration efforts. Studies have demonstrated that Collembola abundance and diversity are closely linked to contamination levels, particularly with polycyclic aromatic hydrocarbons and heavy metals^24^. Other authors have also investigated the use of *Folsomia candida* as a soil quality indicator in high-contamination scenarios of severely degraded mining soils and their remediation^25^. Compared to earthworms, Collembola and other soil mesofauna often exhibit fast responses to soil disturbances, providing early insights into restoration success^26^.

The environmental risks of landfill leachate extend beyond soil, affecting aquatic ecosystems and their biodiversity. Research using model organisms like *Caenorhabditis elegans* highlights leachate’s potential to induce oxidative stress, DNA damage, and alter gene expression related to neurobehavior and antioxidant defenses^27^. Such stress responses, including trehalose synthesis as a protective mechanism, underscore the complexity of leachate toxicity^28^. In natural systems, leachate contamination often disrupts aquatic ecosystems, replacing pollution-sensitive species with more tolerant organisms like sludge worms and chironomids.

Biochar may also affect the springtail *Folsomia candida*. Although there have been few studies on the toxicity of biochars on *Folsomia candida*, it has been shown that biochar can negatively affect soil fauna activity and springtail reproduction^29–31^. In turn, other studies have shown that biochar has no negative effect on the survival and reproduction of springtails, and even increases the number and diversity of springtails^32,33^.

So far, various solutions have been used to treatment leachate from landfills, but solutions are still being sought that will enable their effective application at the place where the leachate is generated. One such solution is the Vegetation-activated sludge process (V-ASP) consists of a combination of constructed wetlands systems (CW) with a conventional activated sludge process (ASP) and is feasible for decentralized wastewater treatment^34–36^. An important element of the treatment system is the presence of plants and the substrate used^37,38^. Commonly used substrates for the treatment of various types of wastewater include zeolites and biochar^39,40^. Biochar can be produced from various types of raw materials, including banana peel waste^41^olive stone, rice husks, wood chips^42^municipal solid and plastic waste^43^cherry kernels^44^and industrial sewage sludge^45^. Due to its properties, including a large specific surface area, high biosorption capacity and ion exchange, biochar is suitable as a substrate in wastewater treatment^46^. Similarly, zeolites are widely used in the removal of inorganic and organic compounds from wastewater due to their large pore surface and microporous structure^40^.

Although toxicity to soil fauna is an important issue, few studies have focused on the effects of leachate on soil fauna. Among soil invertebrates, the use of Collembola *Folsomia candida* as indicators of soil health may be important in the case of leachate contamination. However, to our knowledge, no such studies have been conducted. To date, only a few studies have focused on assessing the toxicity of soil contaminated with landfill leachate; however, these studies have been conducted on earthworms^47,48^.

Therefore, the main objective of this study is to evaluate the survival and reproduction of the model soil-dwelling Collembola *Folsomia candida* in response to exposure to soil spiked with landfill leachate and post-treatment leachate. Leachate treatment was performed in V-ASP systems with two variants of system fills (zeolite, biochar) and in systems with and without plantings. Furthermore, the link between the relative toxicity of leachate and their physicochemical properties for Collembola were assessed in different system variants to identify the main compounds responsible for the observed effects.

We hypothesize that application of raw leachate to soil will inhibit the growth of *F. candida*, whereas treated leachate will not negatively affect to growth inhibition.

## Materials and methods

### Sampling and physicochemical analyses of landfill leachate

The leachate used in the experiment came from an operational landfill located in south-western Poland. The landfill has been operating since 1977, currently covers an area of 14.12 ha and has a total capacity of 2,340,000 m^3^. The landfill has leachate capture and disposal facilities.

Leachate dosed into the systems were collected twice (LL1, LL2). Leachate samples were collected from the leachate retention pond (51°14′14.9"N 16°11′07.0"E).

Leachate samples were collected into 25 L plastic tanks and transported immediately to the laboratory of the Centre for Environmental Quality Analysis of Wrocław University of Environmental and Life Sciences.

Activated sludge for reactors’ inoculation was taken from a nitrification chamber of the municipal wastewater treatment plant (Population equivalent – PE ≈ 1 050 000).

In all series of raw leachate samples and system outflows, basic physicochemical analyses were performed, including: pH, electrical conductivity (EC), Chemical Oxygen Demand (COD) and concentration: total nitrogen (TN), organic nitrogen (ON), ammonium nitrogen (AN), total phosphorus (TP). Moreover, in raw leachate samples (LL1, LL2) and after treatment, i.e. series 2 (after 42 days) and series 4 (after 138 days), an extended range of analyses was performed, including, in addition to the basic parameters: chlorides, sulfates, iron (Fe), manganese (Mn), potassium (K), zinc (Zn), copper (Cu), nickel (Ni), lead (Pb), chromium (Cr) and cadmium (Cd).

Analyses not requiring mineralization were performed within 24 h of sampling^49,50^the others after mineralization.

Below are the results of physicochemical analyses of raw leachate conducted for two series of collected leachate.

**Table 1:** Physicochemical characteristics of Raw landfill leachate^38^.

Parameter	Unit	Raw landfill leachate	
		1 series – LL1	2 series – LL2
pH	[-]	8.3 ± 1.7	8.3 ± 0.2
EC	[µS/cm]	7150 ± 715	6020 ± 120
COD	[mg/l O_2]_	1100 ± 165	620 ± 124
TP	[mg/l]	2.21 ± 0.44	1.85 ± 0.37
TN		154 ± 21	198 ± 40
ON		30.5 ± 6.1	21 ± 4
AN		80 ± 8	108 ± 22
Fe		1.7 ± 0.34	0.496 ± 0.099
Mn		0.217 ± 0.043	0.396 ± 0.079
K		101 ± 20	388 ± 78
Na		169 ± 34	516 ± 103
Ca		34.3 ± 6.9	73.4 ± 14.7
Mg		58 ± 11.6	87.4 ± 17.5
Zn		0.129 ± 0.026	0.08 ± 0.016
Cu		0.036 ± 0.007	0.026 ± 0.005
Ni		0.185 ± 0.037	0.068 ± 0.014
Pb		0.005 ± 0.001	< 0.005
Cr		0.196 ± 0.039	0.62 ± 0.012
Cd		< 0.005	< 0.005
Chlorides		536 ± 79	938 ± 188
Sulfates		139 ± 21	221 ± 44

The analyses of physicochemical properties were carried out using standard methods, according to ISO (International Organization for Standardization) standards. Table S1 presents a list of applied methods of analysis of physicochemical parameters.

The reduction efficiency of selected parameters was calculated according to the following Eq. ^51^:

1 $$R{\text{ }}=~\frac{{{C_{in}} - {C_{out}}}}{{{C_{in}}}}100{\text{ }}\left[ \% \right]$$

where R represents the reduction efficiency of the selected parameters [%],

while C_in_ and C_out_ represent the inflow and outflow concentrations of the selected parameters respectively [mg/l].

### Experimental design

The experiment was carried out in four laboratory polyethylene reactors with a capacity of 10 L each. The experimental system was operated as a SBR on a 24 h cycle consisting five stages: 0.5 h of filling, 3 h of mechanical mixing using a power-driven agitator, 19 h of aeration using a fine-bubble diffuser powered by a compressor, 1 h of activated sludge sedimentation, and 0.5 h of automatic decantation of treated leachate into an effluent collection tank for analysis. All operations were automated, the order in which the elements were activated was regulated by a timer. A basket was placed in each reactor and immersed below the water level (no deeper than 15 cm), according to the methodology proposed by Ref^52^. Depending on the variant, the baskets were filled with zeolite or activated biochar from sunflower husk. Baskets with three plant species selected for the V-ASP system were embedded inside the bed: *Lythrum salicaria L*. (purple loosestrife), *Typha minima* (dwarf bulrush) and *Iris pseudacorus* (yellow flag). Details regarding the experimental design are presented in the publications^38,53^.

In total, 4 reactor variants were prepared with the following fillings made of: zeolite: without plants (system Z) and planted system (system ZP); biochar: without plants (system B) and planted system (system BP).

**Fig. 1:**
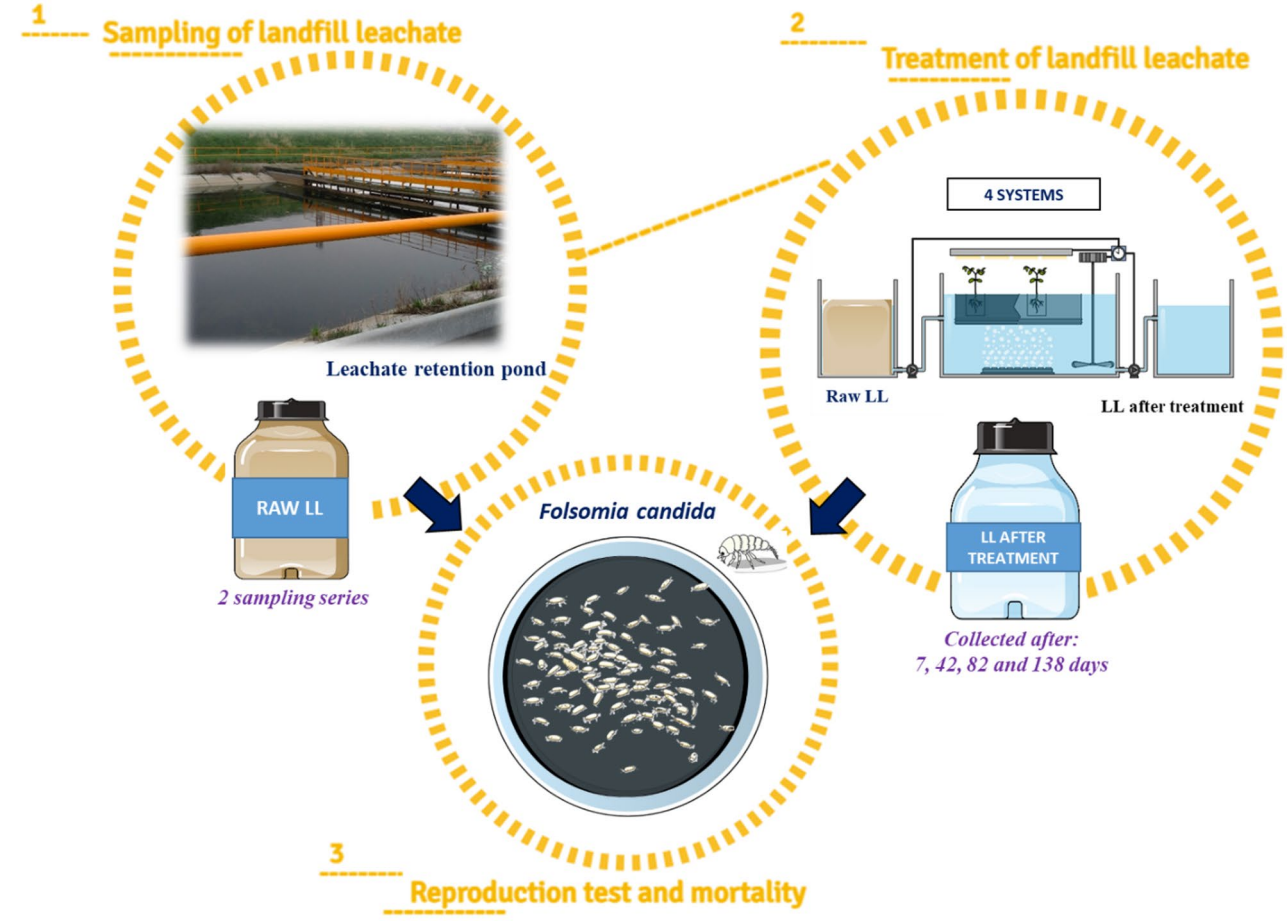
Diagram of the experimental procedure.

The tests used raw leachate (LL1, LL2) and leachate at the reactor outflow collected four times during the experiment (Fig. 1), i.e. after 7 days (series – 1), 42 days (series – 2), 82 days (series – 3) and 138 days (series – 4). The distilled water was the negative control and raw leachate (LL1 and LL2) was used as a positive control.

### Reproduction test and mortality on Collembola

The study was conducted based on Standard Test No. 232: Collembolan Reproduction Test in Soil. A natural soil, collected from an uncultivated area free of pesticide contamination, with the following physicochemical properties was used: textural class: sandy loam, low content (2%) of clay fraction; C_org_: 5.30 g/kg; N total: 0.38 g/kg; pH in H_₂_O: 6.81; pH in Kl: 6.69; density: 1.6 g/mL, CEC (cation exchange capacity): 5 cmol(+)/kg, EC (electrical conductivity: 0.1 ds/m).

Before the experiment, the soil was sieved through a 1 mm mesh. Each experimental variant (18 in total) and the control were replicated six times. One replicate consisted of a 100 mL container filled with 30 g of soil. The soil was moistened with 5 mL of leachate or water (control) according to the experimental design and incubated for three days to ensure uniform hydration. The species *Folsomia candida* aged 10 days was used in the test. Before the experiment, the springtails were cultured on plaster-charcoal substrates. Ten Collembola were introduced into each container along with 2 mg of dry baker’s yeast as food. The containers were sealed with lids featuring perforations to allow gas exchange. The organisms were incubated in a climate chamber at 20 °C, under a 12/12 h light/dark cycle. The incubation period lasted 28 days. Weekly, water losses were replenished, and food was supplemented.

On the 28th day, the samples were flooded with water, causing juvenile individuals to float to the surface. The water was stained with ink, and photographs were taken. Subsequently, adult and juvenile Collembola were counted using ImageJ software. The software processes the images through thresholding and segmentation functions, enabling automatic object detection. Each detected organism was counted, and the results were recorded as numerical data.

Mortality and reproduction are the most commonly used endpoints, and reproduction is considered the single most important function in the life cycle of an organism^54,55^. The mortality and reproduction inhibition of Collembola was calculated using the formula^56^:

2 $${\text{Mortality }}=~\frac{{Number~of~dead~organisms}}{{Number~of~living~organisms~at~the~beginning~of~the~test}}{\text{1}}00~~\left[ \% \right]$$

3 $${\text{Reproduction inhibition }}=~~\frac{{Number~of~juveniles~in~control - EquationNumber~of~juveniles~in~test}}{{Number~of~juveniles~in~control~}}{\text{1}}00~~\left[ \% \right]$$

### Data analysis

The GLM analysis was performed to analyze the differences in the number of adults and juveniles. The factors were the reactor filling (zeolite (Z), biochar (B), zeolite planted (ZP), biochar planted (BP)) in comparison to the control (water) and the raw leachate (LL). The second factor was the timeframe of management (7, 42, 82 and 138 days). The model ft was determined based on the AIC criteria. The analyses were conducted in SAS software, University Edition.

The data analysis was performed using Redundancy Analysis (RDA), which was conducted to assess the relationships between Collembola mortality, juvenile abundance, and selected physicochemical parameters. Only those water quality variables that exhibited a moderate to strong correlation with Collembola mortality or juvenile numbers were included as explanatory variables in the RDA model (Table S2). The RDA was performed using only the data from the exposed conditions, excluding reproduction and mortality data in the control scenario. The Pearson correlation analysis was conducted between Collembola responses and selected physicochemical parameters used for soil contamination. The RDA was carried out using the Canoco 5 software. Additionally, forward selection of variables was performed prior to the RDA. Only variables contributing more than 3% to the explained variation were included in the analysis, and collinearity among explanatory variables was eliminated.

The RDA biplot was used for visualization of the relationships between Collembola responses and the selected physicochemical parameters, and to interpret the impact of each variable on the outcomes.

## Results

### Physicochemical properties of leachate after treatment

The pH value of raw leachate in the first and second sampling series was 8.3 (LL1, LL2 - Table 1), which is typical for leachate from landfills operated for more than 10 years and indicates a mature phase of waste storage^57^. Regardless of the variant, the pH at the reactor outflow ranged from 8.3 to 8.8 (Table 2).

EC in raw leachate ranged from 6020 µS/cm to 7150 µS/cm. EC in the outflow was reduced in most cases, except for 3 series of sampling in the biochar reactor (BP-3). EC reductions ranging from 2.2 to 61.5% were observed in the system effluents, while no reduction was observed in one series (BP-3). TP in raw effluents was low, ranging from 1.85 to 2.21 mg/l (Table 1), and no reduction was observed in most reactors after treatment, regardless of the variant used.

COD in raw leachate ranged from 620 mg/l (LL2) to 1100 mg/l (LL1). In several runs, no COD reduction was noted at the effluent, while in the remaining runs it ranged from 25.5 to 50.9%.

In all reactor variants, very high AN reduction above 99% was achieved throughout the experiment. TN removal, on the other hand, remained at a level from 14.1 to 90.9% (depending on the analyzed period). In several series no ON reduction was noted, while in the remaining cases it ranged up to 98.6% (depending on the period analysed).

However, phosphorus removal remained low throughout the experiment, or no reduction was observed at all. Only once was a higher TP reduction of about 70% noted. Table 2 summarizes the reductions of individual parameters in relation to the values ​​of raw leachate dosed to the systems (focus was only on the series that were used for further analyses on Collembola organisms).

**Table 2:** Reduction (%) of selected physicochemical parameters and pH range after treatment.

Reactor	pH	EC	TP	COD	AN	TN	ON
B -1	8.3–8.6	51.6	-	25.5	99.9	90.9	96.9
B-2	8.3–8.8	12.4	-	43.6	99.8	63.6	31.1
B-3	8.3–8.6	2.2	-	-	99.9	19.2	-
B-4	8.3–8.7	5.3	-	38.1	99.9	39.4	-
Z-1	8.3	58.0	-	46.4	99.7	88.6	96.1
Z-2	8.3–8.4	26.6	19.5	50.9	99.8	50.6	37.7
Z-3	8.3–8.5	10.6	-	-	99.8	23.2	-
Z-4	8.3–8.6	5.3	-	33.5	99.8	38.4	-
ZP -1	8.3–8.5	61.5	69.1	47.3	99.8	87.0	71.1
ZP -2	8.3–8.5	47.4	9.0	50.9	99.9	74.0	60.7
ZP -3	8.3–8.1	21.3	-	-	99.2	39.9	-
ZP -4	8.3–8.7	14.3	-	38.9	99.8	44.4	-
BP -1	8.3–8.2	52.4	-	35.5	99.9	68.5	98.6
BP-2	8.3–8.8	23.1	-	40.9	99.8	62.3	60.7
BP-3	8.3–8.7	*neg.	-	-	99.9	14.1	-
BP-4	8.3–8.8	4.2	-	34.8	99.8	35.4	-

no removal was observed.

In all series of raw leachate samples and system outflows, basic physicochemical analyses were performed, including: pH, electrical conductivity (EC), Chemical Oxygen Demand (COD) and concentration of total nitrogen (TN), organic nitrogen (ON), ammonium nitrogen (AN), total phosphorus (TP). The supplementary materials in Table S2 present the Reduction (%) of physicochemical parameters after treatment - extended scope of analyses. A detailed description of the results of the tests of the physicochemical properties of the leachate before and after treatment is presented in the works^38,53^.

### Responses of Collembola to tested leachate

#### Adults mortality

**Fig. 2:**
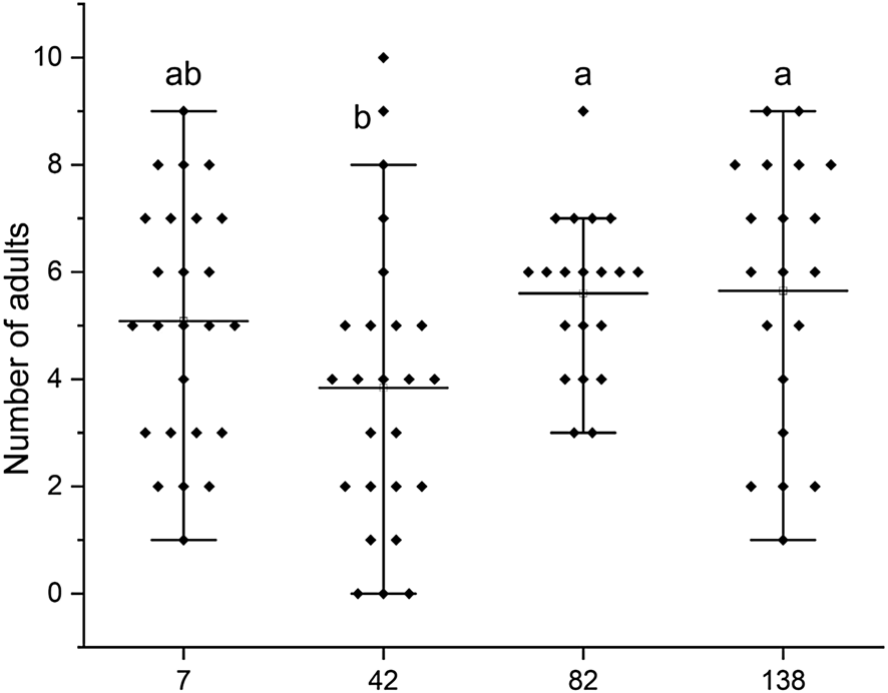
Differences in number of adult in soil contaminated with various reactor fillings, and their interaction effects with time. 7, 42, 82, 138 - The management of leachate timeframe [days], *Different small letters indicate significant differences between treatments.

Regardless of the filling variant used, the leachate collected at the outflow of the systems did not cause mortality in the test organisms. However, a clear effect of leachate incubation time on adult Collembola abundance was observed. The number of adults decreased by 24.4% from day 7 (5.08 individuals) to day 42 (3.84 individuals) (Table 3; Fig. 2).

**Table 3:** Differences in adult abundance in soil contaminated with various reactor fillings and different management timeframes, and their interaction effects after 28 days of incubation.

Factor	DF	R2	Mean square	F	*p*-value
Reactor filling	5	52.25500000	10.45100000	2.04	0.0834
Time	3	51.39526316	17.13175439	3.34	0.0239
Reactor filling*time	10	38.45500000	3.84550000	0.75	0.6751

**Fig. 3:**
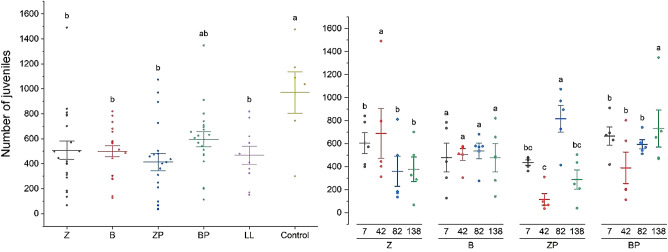
Differences in juvenile abundance in soil contaminated with various reactor fillings, and their interaction effects with time. Systems variants with zeolite: without plants ( Z) and with plant system (ZP); with biochar: without plants (B) and with plant system (BP) and raw leachate (LL). *Different small letters indicate significant differences between treatments.

Table 4 shows the average mortality rate in individual reactors. The mortality did not exceed 20% (Table 4) in the control and the number of juveniles (Fig. 3) was higher than 100 (OECD 232). The test fulfil the validity criteria, the mortality values and juvenile number.

**Table 4:** The mean mortality observed in soil spiked with the selected treatments.

Reactor	Leachate timeframe [days]	Mortality, mean [%]
Z − 1	7	50.0
Z − 2	42	62.0
Z − 3	82	46.0
Z − 4	138	28.0
B − 1	7	48.0
B − 2	42	50.0
B − 3	82	40.0
B − 4	138	50.0
ZP − 1	7	58.0
ZP − 2	42	54.0
ZP − 3	82	36.0
ZP − 4	138	44.0
BP − 1	7	40.0
BP − 2	42	76.0
BP − 3	82	54.0
BP − 4	138	54.0
Raw LL -1	0	66.0
Raw LL -2	0	66.0
Control	0	12.0

The leachate after treatment at the outflow of the systems, regardless of the filling variant used, in all cases affected the reproduction of Collembola. Compared to the control sample (distilled water), which yielded an average of 1,237 juveniles, reproduction was significantly reduced in all treated leachates. Specifically, the number of juveniles decreased by 59.2% with zeolite treatment (504.46 juveniles), 64.9% with biochar (434.29 juveniles), 58.0% with the zeolite–plant system (519.08 juveniles), 49.8% with the biochar–plant system (621.00 juveniles), and 50.5% with the raw leachate system (612.33 juveniles) (Table 5; Fig. 3).

**Table 5:** Differences in juvenile abundance in soil contaminated with various reactor fillings and different management timeframes, and their interaction effects after 28 days of incubation.

Factor	DF	R2	Mean square	F	*p*-value
Reactor filling	6	2123024.26	353837.37	5.8	< 0.0001
Time	3	291475.75	97158.58	1.59	0.1978
Reactor filling*time	9	1789768.75	1.99	3.26	0.0021

Collembola reproduction was slightly reduced in soil contaminated with BP reactor effluent leachate compared to other treatments and raw leachate. The toxicity level of leachate after treatment in other reactors was unchanged from that of raw leachate.

The management timeframe (7, 42, 82 and 138 days) did not significantly impact overall reproductive responses of Collembola. However, interactions between reactor filling and timeframe yielded significant differences in specific cases. The highest reduction in reproduction was observed after the use of leachate after treatment from the ZP reactor, after 42 days, compared to the other periods.

For the redundancy analysis (RDA) as explanatory variables, only the leachate quality parameters were used, which strongly or moderately correlated with Collembola mortality or juveniles’ number (Table 6).

**Table 6:** The correlation coefficient between the Collembola responses (juvenile number and mortality) and the leachate parameters.

Parameter	Juveniles number	Mortality
Juvenilles	1.00	-0.14
Mortality	-0.14	1.00
pH	**-0.32**	0.12
EC	0.04	0.17
TP	0.15	-0.12
COD	0.28	-0.08
AN	-0.10	**0.48**
TN	0.08	-0.01
ON	0.23	**-0.34**
TDS	**0.60**	-0.19
Zn	0.09	**0.32**
Cu	0.11	0.29
Ni	0.22	0.23
Cr	-0.02	**0.33**
Mn	0.16	-0.05
Na	0.18	-0.27
Mg	**0.35**	-0.16
K	0.23	**-0.34**
Ca	-0.01	-0.01
Fe	0.10	**0.54**
Sulfates	0.29	0.17
Chlorides	0.28	0.22

slight correlation: correlation coefficient values from 0.00 to 0.30 (positive or negative); moderate correlation: correlation coefficient values from 0.31 to 0.60 (positive or negative); strong correlation: correlation coefficient values from 0.61 to 1.00 (positive or negative).

The data highlighted in bold indicate moderate correlation between treatments.

The redundancy analysis (RDA) revealed a significant effect of selected leachate quality parameters on both Collembola mortality and juvenile abundance. The first two RDA axes explain the entire variance in the constrained model, with RDA1 accounting for 97.6% and RDA2 for 2.4%, confirming that RDA1 is the primary gradient driving the observed biological responses (Table 7).

**Table 7:** Eigenvalues and variance explained of 2 RDA axes linking Collembola mortality, juveniles number and selected leachate quality parameters.

Axis	Eigenvalue	Cumulative variation explaines
RDA1	0.976	97.6
RDA2	0.024	100.0

**Table 8:** Contribution of environmental variables to variation in collembola mortality and juvenile numbers based on RDA1.

Name	Explains %	Contribution %	pseudo-F	*P*
TDS	60.9	60.9	7.8	0.076
Mg	19.5	19.5	4	0.144
AN	4.8	4.8	1	05555
ON	6.7	6.7	1.6	0.294
pH	3.8	3.8	0.9	0.54
Fe	4.3	4.3	< 0.1	1

**Fig. 4:**
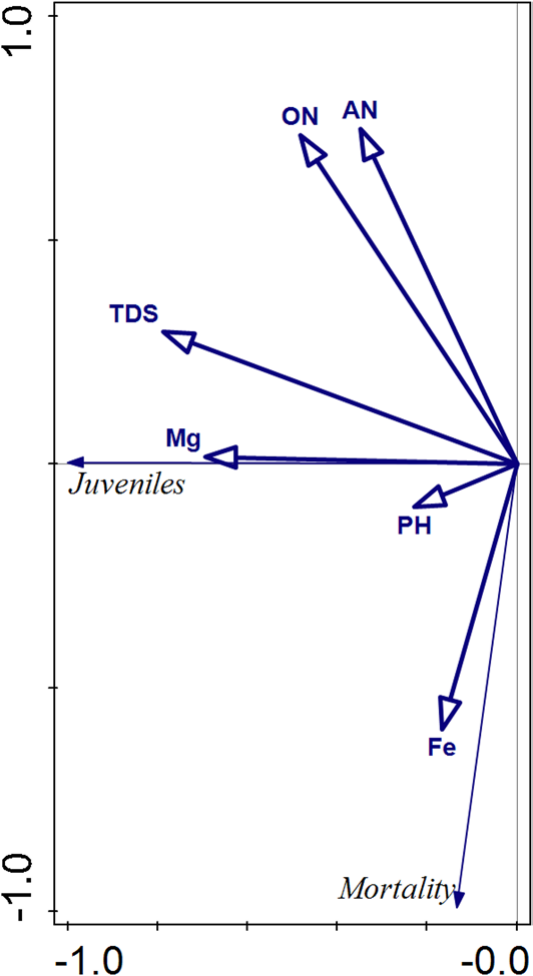
RDA bilplot of relation between Collembola mortality, juveniles number and selected leachate quality parameters.

Among the analyzed parameters, total dissolved solids (TDS) and magnesium (Mg) show a strong positive association with juvenile abundance, suggesting that higher concentrations of these substances may mitigation of the toxicity the development of Collembola juveniles (Fig. 4; Table 8). Similarly, organic nitrogen (ON) and ammonium nitrogen (AN) are also aligned with juvenile abundance, although to a lesser degree.

Conversely, iron (Fe) shows a clear positive association with mortality, indicating that higher Fe concentrations may negatively affect Collembola survival.

## Discussion

Current regulatory approaches for landfill leachate management emphasize physicochemical characterization, focusing on parameters such as pH, conductivity, and specific contaminant concentrations^58^. However, while essential, these parameters fail to capture the cumulative and interactive toxic effects of leachate constituents^59^. In contrast, bioassays offer a more holistic approach, reflecting the integrated biological responses of organisms exposed to the full suite of contaminants present in leachate^60^. Studies conducted to date have highlighted the key importance of species interactions in predicting ecosystem risks^61^. The need for a comprehensive battery of bioassays covering multiple trophic levels has been highlighted as crucial for accurate leachate toxicity assessment^60,62^. Nevertheless, such bioassays are rarely applied to soil invertebrates, including Collembola, despite their essential ecological roles^63^. Collembola is a species often used in ecotoxicological tests, due to its sensitivity to pollutants and changes in soil properties. So far, it has been used, for example, to assess the risks associated with soil contamination by metals^61,64^ or soil amendments^65^ under controlled laboratory conditions. However, few studies have so far included bioassays on soil invertebrates.

Only a few studies have reported that landfill leachates can have a negative effect on the reproduction of *Eisenia fetida* earthworms, resulting in the absence of juveniles^47^. In contrast, other studies have shown that *Eisenia fetida* maintains retains long life and fertility, and the ability to reproduce after exposure to landfill leachates, and even population growth (from 25 individuals in the initial bed to 298 individuals after six months of study)^66^. Other studies have shown that the earthworm *Eisenia fetida* exhibited negative gene expression responses in microcosms exposed to leachate-contaminated^48^,and landfill leachate contamination has been shown to significantly alter soil microbial composition^67,68^.

Studies focusing on aquatic organisms, such as *Lemna minor* (plant), *Daphnia magna* (Cladoceran), and *Thamnocephalus platyurus* (Ostracoda), have consistently demonstrated the high toxicity of landfill leachate, attributing harmful effects to components like ammonia, alkalinity, heavy metals (e.g., lead, cadmium, copper), and persistent organic pollutants^69–71^. These substances reduce growth and reproduction in sensitive species, proving that landfill leachate are highly toxic. Notably, the highest toxicity levels are associated with leachate from landfills receiving mixed domestic and hazardous industrial waste, underscoring the importance of waste composition in determining leachate toxicity profiles^72^.

In recent years, there has been an increasing interest in soil contamination with heavy metals^73^ and soil quality issues, as it is an important element not only for food production but also for maintaining environmental values ​​and ecological balance^74^. Heavy metal contamination poses a serious threat to human health due to the high toxicity, environmental mobility, accumulation in the food chain and lack of biodegradability of heavy metals^75,76^. They can adversely affect soil fauna and flora through soil-microorganism interactions and microbiological processes^77^. The studies conducted so far have confirmed their influence on changes in the metabolic activity of microorganisms, the number and diversity of the soil microbiome^78,79^.

Although no direct studies have assessed landfill leachate toxicity in Collembola, related research on heavy metal toxicity in these organisms gives some information. Lead (Pb), cadmium (Cd), and copper (Cu), have all been shown to adversely affect Collembola survival, growth and reproduction, with lead exhibiting the most severe effects ^80-82^. These impacts are primarily driven by the metals’ interference with essential physiological processes. For instance, heavy metals can disrupt enzymatic activity, impair membrane integrity, and induce oxidative stress by generating reactive oxygen species (ROS). Over time, these disruptions compromise energy metabolism and cellular function, leading to reduced growth and fecundity or increased mortality^80,83^. Given that landfill leachate may contain elevated concentrations of these metals, it is highly plausible that similar negative impacts would be observed in Collembola populations exposed to contaminated soils. In this study, redundancy analysis (RDA) revealed that iron (Fe) was positively associated with increased *Folsomia candida* mortality, suggesting that elevated Fe levels may contribute to toxicity, potentially through similar oxidative and metabolic pathways. Such an effect may occur, where the elevated pH of the leachates (8.3–8.8) could increase soil pH and influence iron toxicity to Collembola. According to the study by Beaulieu et al., collembola species adapted to more acidic soils tend to be more sensitive to iron under neutral to alkaline conditions, likely due to changes in iron availability and speciation^84^. In contrast, total dissolved solids (TDS) and organic nitrogen (ON) were positively correlated with juvenile abundance, indicating that certain soil constituents may mitigate toxicity or provide nutritional benefits that enhance juvenile development. These findings are consistent with previous studies demonstrating the negative impact of heavy metals on Collembola. Additionally, studies on the effect of soil metal contamination on *Folsomia candida* have shown negative relationships between the survival and reproduction of adults and the concentration of Zn^85^. Furthermore, copper and zinc reduced survival and reproduction in *Sinella curviseta* (Collembola)^86^and dietary exposure to heavy metals affected growth, reproduction, and avoidance behavior in *Folsomia candida* (Collembola), with cadmium identified as the most toxic^87^. The positive association of TDS and ON with juvenile numbers may reflect the importance of organic matter in supporting F. candida development. Organic matter has the ability to bind soluble heavy metals and organic contaminants, decreasing their availability to soil organisms. Therefore, higher levels of total dissolved solids (TDS) may help lessen the toxicity caused by adding leachates to the soil. Additionally, the origin of the organic material—such as more resistant compounds derived from plant sources associated with increased organic nitrogen (ON)—can result in a slower release of potentially harmful substances into the soil environment^88,89^. Other authors have observed that appropriate concentrations of organic matter in soil are crucial for the growth and development of *F. candida*^90^. Organic matter enhances fungal growth, which serves as the primary food source for this species^91^.

Also soil properties, such as pH and organic matter content, are known to significantly influence metal bioavailability and toxicity, with low organic matter and acidic pH increasing cadmium sensitivity^92^. In this context, the observed effects of TDS and ON on juvenile numbers may also reflect indirect interactions, potentially modulating heavy metal toxicity.

## Conclusions

1. Raw and treated leachate, regardless of treatment variant and period, affected Collembola reproduction in all cases. In most cases, fewer juveniles were observed compared to the control group. However, the leachate after treatment from the remaining reactors showed similar toxicity levels to the raw leachate.
2. Redundancy analysis (RDA) identified iron (Fe) as a key factor associated with increased Collembola mortality, suggesting that elevated Fe concentrations may play a significant role in leachate toxicity. In contrast, total dissolved solids (TDS) and organic nitrogen (ON) were positively associated with juvenile abundance, indicating that these parameters may support or indirectly enhance juvenile development - potentially through improved soil conditions or food availability.
3. The results obtained show that these systems have the potential to remove contaminants from landfill leachate. However, the effectiveness of the proposed solutions is not satisfactory for all contaminants present in the leachate. This residual contamination could pose risks to soil organisms in the environment. If heavy metals and other toxic substances persist in the leachate, they may harm soil fauna and disrupt overall soil health. All these problems require further, extensive research.

## Electronic supplementary material

Below is the link to the electronic supplementary material.

Supplementary Material 1

## Data Availability

The datasets used and/or analysed during the current study available from the corresponding author on reasonable request.
