# Supplementary material for: The effect of landfill leachate treatment on ecotoxicological properties of Folsomia candida, with a focus on soil contamination risks
Source: Sci Rep. 2025 Jul 2;15:22688. doi: 10.1038/s41598-025-07799-5 (PMC12215456; doi:10.1038/s41598-025-07799-5)
Supplement: Supplementary file 1 — Supplementary Material 1 [file 41598_2025_7799_MOESM1_ESM.docx]

**Table S1.** Methods for the determination of landfill leachate contamination indicators

| **Compounds** | **Unit** | **Name of the method** | **Standard** |
| --- | --- | --- | --- |
| pH | - | Potentiometric method | PN-EN ISO 10523:2012 |
| EC | (μS/cm) | Conductometric method | PN-EN 27888:1999 |
| COD | (mg O_2_/l) | Miniaturized method using tight test tubes | PN-ISO 15705:2005 |
| TN | (mg/l) | Spectrophotometric method | PBM-14 EDITION 3 DATED 03/10/2022 BASED ON MERCK TEST 1.00613 |
| ON |  | Computational method | PBM-01 issue 3 of May 10, 2022 based on Merck test 1.14752 |
| AN |  | Spectrophotometric method | PBM-01 issue 3 of May 10, 2022 based on Merck test 1.14752 |
| TP, K, Na, Ca, Mg, Fe, Mn, Zn, Cu, Ni, Pb, Cr, Cd |  | Atomic emission spectrometry method  excitation in inductively coupled plasma | PBW-11 issue 6 of September 9, 2022 |
| Chlorides, Sulfates |  | Ion chromatography method | PN-EN ISO 10304-1:2009 |

**Table S2.** Reduction (%) of physicochemical parameters after treatment - extended scope of analyses.

| Parameter  [mg/l] | Reactor | | | | | | | |
| --- | --- | --- | --- | --- | --- | --- | --- | --- |
|  | Z - 2 | Z - 4 | B - 2 | B - 4 | ZP - 2 | ZP - 4 | BP - 2 | BP - 4 |
| Zn | 63.6 | *neg. | *neg. | 8.8 | 68.2 | *neg. | *neg. | *neg. |
| Pb | 50.0 | 0.0 | 50.0 | 0.0 | 50.0 | 0.0 | *neg. | 0.0 |
| Cd | 0.0 | 0.0 | 0.0 | 0.0 | 0.0 | 0.0 | 0.0 | 0.0 |
| Cu | 58.3 | *neg. | 36.1 | *neg. | 72.2 | *neg. | *neg. | *neg. |
| Ni | 59.5 | *neg. | 56.2 | *neg. | 69.7 | *neg. | 58.9 | *neg. |
| Cr | 77.0 | 83.1 | 74.0 | 82.9 | 79.1 | 83.1 | 75.0 | 82.6 |
| Mn | 71.9 | 36.4 | 78.3 | 38.9 | 89.9 | 52.8 | 58.5 | 38.4 |
| Na | *neg. | *neg. | *neg. | *neg. | *neg. | *neg. | *neg. | *neg. |
| Mg | *neg. | *neg. | *neg. | 2.2 | *neg. | *neg. | *neg. | *neg. |
| K | *neg. | 35.8 | *neg. | 9.5 | 13.3 | 31.4 | *neg. | 11.1 |
| Ca | *neg. | *neg. | *neg. | 3.5 | *neg. | *neg. | *neg. | *neg. |
| Fe | 91.5 | 21.0 | 48.6 | *neg. | 84.4 | *neg. | *neg. | *neg. |
| Sulfates | *neg. | 17.2 | *neg. | 11.8 | *neg. | 20.4 | *neg. | 17.6 |
| Chlorides | *neg. | 22.4 | *neg. | *neg. | *neg. | 8.3 | *neg. | 6.1 |
